# Cancer Stemness and Dedifferentiation in Anaplastic Thyroid Carcinoma: Insights into a Multigenic, Microenvironmental Network and the Role of CD44

**DOI:** 10.3390/biomedicines14020453

**Published:** 2026-02-18

**Authors:** Benny Mosoane, Brandon S. Jackson, Michelle McCabe, Tebogo Marutha, Zodwa Dlamini

**Affiliations:** 1 Department of Anatomical Pathology, National Health Laboratory Services, Tshwane Academic Division, University of Pretoria, Pretoria 0001, South Africa; michelle.mccabe@nhls.ac.za; 2 Department of General Surgery, Breast and Endocrine Unit, University of Pretoria, Kalafong Provincial Tertiary Hospital, Pretoria 0001, South Africa; brandon.jackson@up.ac.za; 3 SAMRC Precision Oncology Research Unit (PORU), DSI/NRF SARChI Chair in Precision Oncology and Cancer Prevention (POCP), Pan African Cancer Research Institute (PACRI), University of Pretoria, Hatfield 0028, South Africa; tebogo.marutha@up.ac.za; 4 Wolfson Wohl Cancer Research Centre, School of Cancer Sciences, University of Glasgow, Garscube Estate, Switchback Road, Glasgow G61 1QH, UK

**Keywords:** cancer stem cells, anaplastic thyroid carcinoma, dedifferentiation, CD44

## Abstract

Anaplastic thyroid carcinoma (ATC) is an aggressive and lethal malignancy that carries a poor prognosis. Moreover, there are limited therapeutic options for managing ATC. There is increasing evidence that implicates the role of cancer stem cells (CSCs) in the processes of dedifferentiation in the progression, therapeutic resistance, and metastatic potential of ATC. In this review, we integrate the molecular and cellular insights into the CSCs paradigm in ATC to highlight the role of stemness-associated markers that include CD44, CD133, and ALDH1. We put special emphasis on the role of CD44 and its variant isoforms (CD44v), which play a role in the interface of cancer stemness, tumour microenvironment crosstalk, modulation of epithelial–mesenchymal transition (EMT), chemoresistance, and metastasis. The contribution of signalling pathways (PI3K/AKT/mTOR, MAPK, Notch, Wnt/β-catenin, and Hedgehog) to hypoxia, cancer-associated fibroblasts (CAFs), and tumour-associated macrophages (TAMs) in sustaining CSC niches will be discussed. The review explores advances in molecular diagnostics, imaging technologies, and targeted therapeutic strategies with the potential to disrupt CSC-driven tumour maintenance. Through integration of multigenic, epigenetic, and microenvironmental perspectives, this review highlights the potential necessity of CSC-targeted and combination therapies to improve disease outcomes in ATC.

## 1. Introduction

Anaplastic thyroid carcinoma (ATC) comprises fewer than 2% of thyroid cancers; however, it accounts for a disproportionately high percentage of thyroid cancer-related mortality [1]. The disease is common in women, with peak incidence in individuals who are 60 years and older [2]. ATC presents as a rapidly enlarging fixed neck mass associated with dysphagia, dyspnoea, hoarseness of voice, respiratory distress, recurrent laryngeal nerve palsy, ulceration, and chyle fistula formation [3,4]. These aggressive features contribute to limited treatment options, especially if the diagnosis is delayed [5]. Despite its rarity, ATC represents the terminal end of thyroid cancer progression and provides a unique biological model for studying dedifferentiation, cancer stemness, and treatment resistance, which are the processes that are also relevant to disease progression in more common thyroid carcinomas.

The higher incidence of ATC observed in certain regions has been linked to the underlying prevalence of follicular thyroid carcinoma (FTC), suggesting that FTC may be more prone to dedifferentiation into the anaplastic subtype compared to papillary thyroid carcinoma (PTC) [6,7]. FTC tends to occur more frequently in iodine-deficient areas, where chronic elevation of thyroid-stimulating hormone (TSH) and nodular hyperplasia may create a microenvironment conducive to malignant transformation and progression [8].

In a recent multi-institutional study on thyroid malignancies in South Africa, ATC accounted for 1.5% of 464 histologically confirmed cases. Notably, there was a significant geographic variation in distribution, with nearly half of all ATC diagnoses reported from Limpopo Province, South Africa (3 out of 7 cases), suggesting potential regional or demographic influences on disease prevalence [9]. Beyond Limpopo Province, the multi-institutional nature of the South African cohort suggests that ATC cases are captured predominantly at tertiary referral centres, with regional differences likely reflecting disparities in healthcare infrastructure rather than true geographic variation in disease biology. Variability in access to early thyroid evaluation, fine-needle aspiration cytology, surgical services, and radioiodine therapy may influence both stage at diagnosis and the likelihood of dedifferentiation from well-differentiated thyroid carcinoma to ATC. Similar patterns have been reported in other low- and middle-income country settings, such as Indonesia and Sudan, where single-institution studies indicate that patients with aggressive thyroid cancers frequently present at advanced stages, have limited access to multimodal therapy, and experience extremely poor survival outcomes [10,11]. These observations highlight the influence of healthcare infrastructure, referral pathways, and delayed diagnosis in shaping apparent disease distribution, rather than intrinsic tumour biology alone.

ATC is universally recognised for its exceptionally poor prognosis, with limited improvements in long-term survival. Median overall survival at one year is approximately 20%, and five-year survival remains below 7% [12,13]. While high-income countries may offer access to more advanced therapeutic interventions, overall outcomes remain dismal. In low- and middle-income countries (LMICs), socioeconomic constraints such as delayed diagnosis, limited access to specialised oncology care, and under-resourced healthcare systems may further worsen survival outcomes [14]. Reflecting its aggressive clinical behaviour, the American Joint Committee on Cancer (AJCC) uniformly categorises all ATC cases as stage IV at the time of diagnosis [15].

Cancer stem cells (CSCs) represent a distinct subpopulation within tumours characterised by their capacity for self-renewal, sustained proliferation, and the ability to initiate metastasis [16]. These cells have been implicated in key oncogenic processes including tumour initiation, progression, therapy resistance, and disease recurrence. In thyroid cancers, the presence of thyroid cancer stem cells (TCSCs) offers a compelling explanation for the variable clinical behaviour and resistance to conventional treatments [17]. Advances in stem cell biology have facilitated the identification and functional characterisation of TCSCs across various thyroid tumour subtypes, enhancing our understanding of thyroid tumorigenesis.

Experimental evidence supports the existence of TCSCs, including their ability to form thyrospheres in vitro and to reproduce tumour phenotypes in immunocompromised mouse models [17]. During epithelial–mesenchymal transition (EMT), TCSCs undergo phenotypic changes such as loss of cell polarity and adhesion, driven in part by upregulation of transcription factors like Snail and suppression of E-cadherin, promoting an invasive, migratory phenotype [18].

ATC is thought to arise through a stepwise process of dedifferentiation from pre-existing differentiated thyroid cancers (PTC or FTC). Early malignant cells progressively acquire driver mutations and lose differentiation markers, with ATC representing the most aggressive and least differentiated stage, often expressing oncofoetal proteins such as fibronectin [19]. Recent multi-region sequencing studies demonstrate that ATCs share a genomic origin with co-occurring differentiated thyroid carcinomas and emerge via the accumulation of characteristic clonal driver mutations, supporting a stepwise model of ATC development [20].

Several molecular markers have been used to identify TCSCs, including CD44, CD133, and aldehyde dehydrogenase (ALDH) [21,22]. Among these, CD44, particularly its variant isoforms (CD44v) has emerged as a key mediator of cancer stemness and tumour microenvironmental interaction, implicated in promoting self-renewal, metastatic potential, and therapeutic resistance within the multigenic landscape of ATC [23]. ALDH-positive cells isolated from ATC have shown enhanced migratory capacity compared to their counterparts in PTC and FTC, correlating with elevated c-MET and AKT signalling [17]. Additionally, CD133-positive ATC cell lines have demonstrated tumourigenicity in NOD/SCID mice, and primary TCSCs isolated based on ALDH activity not only formed thyrospheres but also replicated tumour behaviour in murine thyroid gland models [24].

This review aims to synthesise emerging evidence linking cancer stemness and dedifferentiation to the pathogenesis and clinical behaviour of anaplastic thyroid carcinoma. Unlike our previous review, which focused broadly on CD44 variant expression across follicular cell-derived thyroid cancers and its role in multidrug resistance, the present article specifically centres on ATC as a biological model of extreme dedifferentiation and stemness. By integrating data on tumour evolution, cancer stem cell biology, and the tumour microenvironment, this review highlights how CD44 and related stemness-associated pathways contribute to aggressiveness, therapeutic resistance, and poor clinical outcomes in ATC. This ATC-focused, stemness-driven perspective provides additional biological and translational insights beyond CD44 expression alone.

## 2. Molecular Drivers of Dedifferentiation in ATC

### 2.1. Common Genetic Alterations

A thorough understanding of the molecular alterations underlying ATC is essential for the development of effective therapeutic strategies. ATC’s aggressive phenotype is driven by a range of genetic abnormalities that promote uncontrolled growth, dedifferentiation, and invasiveness. Among the most frequently implicated mutations are those affecting the MAPK and PI3K-AKT pathways, as well as genes regulating chromatin remodelling and genomic stability. Key recurrent mutations include those in *BRAF*, *TP53*, *PIK3CA*, *PTEN*, *RAS*, *TERT* promoter, *EIF1AX*, *RET*, and components of the switch/sucrose nonfermenting (*SWI/SNF*) complex, which together contribute to the tumour’s aggressive biology and resistance to therapy [25].

Recent comprehensive genomic profiling studies have further elucidated the mutation landscape of ATC, highlighting the clinical relevance of certain driver mutations. Lai et al. (2020) demonstrated that RAS and PIK3CA mutations serve as negative predictors of survival in ATC patients, underscoring their contribution not only to tumour progression but also to poor clinical outcomes [26]. Their study emphasized the importance of these mutations in risk stratification and as potential therapeutic targets.

Similarly, targeted next-generation sequencing analyses by Latteyer et al. (2016) revealed high frequencies of mutations in TP53, RAS, BRAF, ALK, and NF1 in ATC samples, reinforcing the heterogeneous genetic profile of this tumour type and its aggressive biology [27]. The identification of concurrent mutations across these oncogenes and tumour suppressors provides insight into the complex molecular mechanisms driving tumourigenesis and therapy resistance. Khan et al. (2019) reported unique mutation patterns specific to ATC through comprehensive genomic profiling, highlighting recurrent alterations in TP53, TERT promoter, and PI3K/AKT pathway genes. Their work emphasized the molecular divergence of ATC from differentiated thyroid carcinomas and suggested potential avenues for personalised treatment based on mutational signatures [28].

Furthermore, Yoo et al. (2019) integrated genomic and transcriptomic data to characterize aggressive thyroid cancers, identifying key mutations and expression profiles associated with disease progression. Their findings highlighted the synergistic effects of mutations in RAS, BRAF, and TERT promoter genes, contributing to dedifferentiation and metastasis in ATC [29]. This integrative approach underscored the necessity of combined molecular analyses to fully understand ATC pathogenesis and to develop effective targeted therapies.

Collectively, these studies augment the current understanding of ATC molecular drivers, highlighting the prognostic and therapeutic implications of RAS, PIK3CA, TP53, and TERT promoter mutations, among others. Incorporating these findings strengthens the molecular framework necessary for advancing precision oncology in this aggressive thyroid malignancy.

#### 2.1.1. BRAF Mutations in ATC

The *BRAF* gene, located on chromosome 7q34, encodes a serine/threonine kinase that plays a critical role in the mitogen-activated protein kinase (MAPK) signalling cascade, a pathway essential for regulating cellular proliferation, differentiation, and survival [30]. Oncogenic mutations in *BRAF*, particularly the V600E substitution, lead to constitutive activation of the MAPK pathway, bypassing normal feedback inhibition and resulting in persistent ERK signalling [31,32]. Emerging evidence indicates that *BRAF V600E* mutations also contribute to upregulation of CD44, a cancer stem cell marker, thereby promoting tumour plasticity and stemness traits in thyroid cancer cells [33,34,35]. This continuous activation promotes uncontrolled tumour growth and contributes to the dedifferentiated, aggressive phenotype characteristic of ATC [25].

The *BRAF V600E* mutation causes a significant increase in kinase activity estimated at over 500-fold compared to wild-type *BRAF*, which drives oncogenesis independent of upstream RAS signalling [30]. While not exclusive to ATC and seen more commonly in PTC, *BRAF* mutations have been reported in approximately 10–50% of ATC cases, often in tumours arising from pre-existing differentiated thyroid carcinomas [36,37,38].

Clinically, the presence of a *BRAF V600E* mutation in ATC has been linked to poorer overall survival, although it also provides a potential therapeutic target [37]. The combination of dabrafenib (a BRAF inhibitor) and trametinib (a MEK inhibitor) has demonstrated significant clinical benefit in patients with *BRAF V600E*-mutant ATC, offering improved response rates and tolerability compared to traditional cytotoxic chemotherapy [39]. This targeted approach exemplifies the promise of precision oncology in a tumour type historically associated with dismal outcomes.

#### 2.1.2. TERT Promoter Mutations in ATC

Telomerase is a ribonucleoprotein enzyme complex responsible for elongating telomeres, a tandem TTAGGG nucleotide that repeats at the ends of chromosomes. Its catalytic subunit, telomerase reverse transcriptase (TERT), plays a central role in telomerase function, supported by an RNA template and accessory proteins [40,41]. While telomerase is active in germline and stem cells, its expression is repressed in most somatic cells, where telomeres progressively shorten with each cell division. Upon reaching a critical length, this attrition induces replicative senescence [42,43]. However, telomerase reactivation in cancer cells allows continued proliferation and contributes to oncogenic immortality [44,45]. Among the telomerase components, *TERT* reactivation rather than upregulation of the RNA subunit is considered the key oncogenic driver [46,47].

Two recurrent hotspot mutations in the *TERT* promoter region, C228T and C250T, have been widely characterised in thyroid malignancies. These mutations create novel binding sites for E26 transformation-specific (ETS) transcription factors, thereby enhancing TERT expression [48]. While rare in benign thyroid lesions and non-invasive follicular thyroid neoplasms, they are frequently encountered in aggressive thyroid cancers. Reported prevalences of *TERT* promoter mutations are approximately 12–14% in PTC, 15.7% in FTC, 33.8% in poorly differentiated thyroid carcinoma (PDTC), and up to 55–70% in ATC [48,49,50,51]. The C228T mutation predominates over C250T across all histological subtypes, accounting for over 80% of TERT-mutant cases in PTC and ATC [41].

The co-occurrence of *TERT* promoter mutations with other driver mutations, particularly *BRAFV600E* has been associated with more aggressive tumour phenotypes, enhanced metastatic potential, and worse clinical outcomes [52]. This molecular synergy has been observed in ATC and in aggressive subtypes of differentiated thyroid carcinoma such as the diffuse sclerosing variant of PTC. Yoo et al. (2019) employed integrative genomic and transcriptomic analyses to demonstrate that TERT promoter mutations frequently co-occur with RAS and *BRAF* mutations, synergistically promoting dedifferentiation and metastatic progression in aggressive thyroid cancers including ATC [29]. These findings provide important molecular context for the aggressive phenotype and poor prognosis associated with *TERT* promoter mutations. Importantly, TERT promoter mutations are largely absent in benign thyroid neoplasms, medullary thyroid carcinoma, and non-invasive follicular thyroid neoplasms, supporting their utility as a marker of malignant potential and dedifferentiation [53,54].

#### 2.1.3. RAS, RET and PI3K/AKT Pathway Alterations in ATC

The RAS family of oncogenes (*HRAS*, *NRAS*, and *KRAS*) plays a critical role in the regulation of two major intracellular signalling cascades: the MAPK (RAS-RAF-MEK-ERK) and PI3K/AKT pathways. These pathways mediate cell proliferation, survival, and differentiation. Activating mutations in RAS genes have been identified in both benign and malignant thyroid neoplasms, including follicular adenomas and carcinomas, as well as ATC [55]. In ATC, *RAS* mutations are variably reported, with frequencies ranging from 6% up to 50% depending on the cohort and molecular detection method [56,57,58]. Lai et al. (2020) further identified RAS and PIK3CA mutations as significant negative predictors of survival in ATC patients, highlighting the prognostic value of these alterations alongside their known roles in activating proliferative signalling pathways [26]. These mutations contribute to the activation of proliferative and anti-apoptotic pathways that drive tumour progression [59,60].

The RET proto-oncogene encodes a receptor tyrosine kinase that is classically associated with familial medullary thyroid carcinoma and sporadic medullary carcinoma via activating point mutations, and with PTC through RET/PTC rearrangements [55]. Although RET is not a predominant driver in ATC, amplification of the RET locus has been observed in a subset of cases. In a study by Nakashima et al. (2007), RET amplification was reported in seven ATC samples, of which three also demonstrated increased TP53 expression by immunohistochemistry, suggesting a potential link between TP53-related genomic instability and secondary RET activation [61].

The PI3K/AKT signalling pathway is a key regulator of cellular metabolism, growth, and survival [62]. Its activation in ATC is frequently mediated by genetic alterations in components such as *PIK3CA*, *AKT1*, and *PTEN* [55]. *PIK3CA* encodes the p110α catalytic subunit of PI3K and is located on chromosome 3q26.3. Activating mutations in *PIK3CA* occur in approximately 12–23% of ATCs [58,63,64,65], while copy number gains have been reported in up to 61% of cases. In contrast, PIK3CA mutations are infrequent in differentiated thyroid carcinomas, appearing in only 6–13% of FTCs and 1–3% of PTCs [63,66,67].

Downstream activation of AKT1 is a consistent feature in ATC, observed in 85–93% of tumours [58,63], contributing to increased tumour cell survival and resistance to apoptosis. Concurrently, ERK activation, a hallmark of MAPK signalling has been reported in approximately 65% of ATC cases [58], highlighting the frequent co-activation of both MAPK and PI3K pathways. Loss-of-function mutations or deletions in PTEN, a tumour suppressor that negatively regulates PI3K signalling, have been identified in up to 17% of ATCs [65], further enhancing pathway activation and tumour aggressiveness.

#### 2.1.4. Wnt/β-Catenin Pathway Alterations in ATC

The Wnt/β-catenin signalling cascade plays a fundamental role in cell fate determination, stem cell maintenance, and tissue regeneration. Aberrant activation of this pathway is increasingly implicated in the pathogenesis of ATC [68]. In the canonical Wnt pathway, extracellular Wnt ligands bind a membrane-bound receptor complex comprising Frizzled (FZD) receptors and low-density lipoprotein receptor-related proteins (LRP5/6), leading to inhibition of the β-catenin destruction complex. This complex includes key intracellular regulators such as glycogen synthase kinase 3β (GSK3β), casein kinase 1α (CK1α), axis inhibition protein (AXIN), and adenomatous polyposis coli (APC). In the absence of Wnt ligands, β-catenin is phosphorylated by this complex, tagged for ubiquitination, and degraded via the proteasome, preventing transcription of Wnt target genes [69,70]. Ligand binding disrupts this complex, allowing β-catenin to accumulate and translocate to the nucleus, where it activates target genes including *cyclin D1* and *c-MYC* [70].

In ATC, dysregulation of this pathway has been substantiated by genomic and protein expression studies. A Japanese cohort study revealed mutation frequencies of 4.5% in *CTNNB1* (β-catenin), 9.0% in ATC, and a striking 81.8% in *AXIN1*, accompanied by overexpression of Wnt target genes *cyclin D1* (27.3%) and c-MYC (59.1%) [71]. However, these findings are not supported by most subsequent studies, particularly those using NGS. This discrepancy likely reflects methodological differences that were not employed, and the fact that ATCs typically have low tumour purity, which complicates mutation detection. More recently, NGS-based analyses have shown CTNNB1 and AXIN1 alterations to be rare in ATC. Notably, large-scale and multi-region genomic profiling studies reported low mutation frequencies of these genes in ATC [20,72]. Despite this, transcriptional and proteomic data suggest that ATCs can remain functionally dependent on Wnt/β-catenin signalling, indicating that non-mutational mechanisms, such as pathway activation via upstream or epigenetic events, may drive this dependency [72]. This functional reliance, despite the rarity of canonical pathway mutations, highlights the importance of exploring alternative mechanisms of Wnt/β-catenin activation in ATC progression and therapy resistance.

Other evidence supports functional Wnt/β-catenin pathway involvement in ATC progression. The abnormal spindle-like microcephaly-associated protein (ASPM), which regulates mitotic spindle orientation, was shown to promote ATC progression by enhancing Wnt/β-catenin signalling activity [73].

Therapeutic resistance in ATC has also been associated with Wnt pathway activation. Notably, resistance to artemisinin was correlated with Wnt hyperactivation and was reversed by the Wnt inhibitor pyrvinium pamoate [74]. Experimental therapeutics targeting this pathway have shown preliminary promise. A conditionally replicative adenovirus (HILMI), engineered to exploit Wnt pathway activation, demonstrated selective cytotoxicity in ATC models [75]. Additionally, ellagic acid was reported to inhibit ATC cell growth in vitro by simultaneously impeding the Wnt/β-catenin and PI3K/AKT pathways [76].

Although earlier reports suggested frequent *AXIN1* mutations, these findings are not corroborated by more recent genomic studies using NGS [71,77]. In contrast, *APC*, another component of the destruction complex, is rarely mutated in ATC [71]. Taken together, the available evidence suggests that direct genetic alterations of Wnt effectors are uncommon in ATC, yet the pathway appears to be aberrantly activated via alternative mechanisms.

Despite these findings, a comprehensive understanding of the Wnt pathway’s functional role in ATC pathogenesis and therapeutic resistance remains limited. Further research is warranted to elucidate context-specific pathway interactions and to evaluate the clinical efficacy of Wnt-targeted therapies.

#### 2.1.5. Overview of TP53 in ATC Tumourigenesis

*TP53* encodes the p53 protein, a central tumour suppressor involved in safeguarding genomic integrity. Through transcriptional regulation of genes such *as CDKN1A* (*p21*), *p53* mediates critical cellular responses, including DNA repair, cell cycle arrest, senescence, and apoptosis [78]. In ATC, *TP53* is the most frequently mutated gene, with mutation rates ranging between 17% and 80%, and an average prevalence of approximately 44% across studies [25,78,79]. Targeted sequencing studies by Latteyer et al. (2016) and Khan et al. (2019) confirmed TP53 as the most frequently mutated gene in ATC, with mutation rates exceeding 50% in some cohorts. These mutations often coexist with alterations in the TERT promoter and PI3K/AKT pathway genes, potentially driving enhanced tumour aggressiveness and resistance to conventional therapies [27,28]. Such co-occurrence reflects the complex molecular heterogeneity of ATC and reinforces the central role of TP53 disruption in anaplastic transformation. These mutations play a key role in the progression from well-differentiated thyroid carcinoma (WDTC) to the undifferentiated, highly aggressive ATC phenotype.

Missense mutations in *TP53*, especially at “hot spot” codons like R175H and R273H, not only result in loss of normal tumour-suppressive function but also confer oncogenic properties [80]. Mutant p53 proteins may promote tumour growth by enhancing cellular proliferation, survival, angiogenesis, and metastatic potential [81]. This gain-of-function activity marks a shift from passive tumour suppressor loss to active oncogenesis in ATC.

The protein mouse double minute 2 homolog (MDM2), encoded by the *MDM2* gene, is a major negative regulator of p53. It promotes p53 degradation via the ubiquitin–proteasome pathway. Studies comparing anaplastic tumours and their differentiated precursors indicate higher expression of both p53 and MDM2 in ATCs, suggesting p53 dysregulation is often accompanied by MDM2 upregulation [81,82,83,84]. However, isolated MDM2 overexpression without p53 dysregulation appears insufficient to drive anaplastic transformation [85].

Mutant *p53* has been shown to interfere with the transcriptional activity of p63, a member of the p53 protein family. Of the two main isoforms of p63, TAp63 induces apoptosis and suppresses metastasis, while ΔNp63 promotes cell survival and tumour progression [80]. p63 regulates target genes such as *SHARP1*, a transcription factor involved in inhibiting tumour cell migration and invasion. In ATC, mutant p53 may block TAp63 function, leading to downregulation of SHARP1 and enhanced tumour aggressiveness [86].

Activating transcription factor 3 (ATF3), a member of the ATF/CREB family, has emerged as a modulator of mutant p53 activity [87]. ATF3 can bind to mutant p53, disrupt its oncogenic conformation, and restore the tumour-suppressive functions of p63, thereby reactivating SHARP1 expression [88]. Functional studies in the chemoresistant 8305C thyroid cancer cell line harbouring the R273C p53 mutation have shown that ATF3 overexpression reduces cell proliferation, induces apoptosis, and inhibits migration [80,88]. This suggests a therapeutic potential for ATF3 in targeting TP53-mutated ATC.

Although ATF3 has demonstrated tumour-suppressive roles in glioblastoma, bladder, colon, and thyroid cancers, other studies have shown its oncogenic role in breast cancer and cutaneous squamous cell carcinoma [80,89,90,91,92]. Thus, the role of ATF3 in tumourigenesis appears to be context-dependent. Nevertheless, in the setting of ATC, especially in tumours with mutant p53, ATF3 functions as a promising suppressor of tumour progression.

Disruption of the TP53 pathway is a hallmark of ATC, contributing to its rapid progression and therapeutic resistance. The intricate interactions between mutant *p53*, *MDM2*, *p63*, *SHARP1*, and *ATF3* illustrate the multifaceted mechanisms driving tumour aggressiveness. Targeting this network, particularly by enhancing ATF3 activity or restoring p63 function, may offer novel therapeutic avenues for managing p53-mutant ATC.

### 2.2. Epigenetic Alterations

Epigenetic changes, especially mutations in chromatin remodelling complexes, play a major role in the dedifferentiation and aggressive behaviour of ATC. Components of the *SWI/SNF* complex, including *ARID1A*, *ARID2*, *SMARCB1*, and *SMARCA4*, are frequently mutated in approximately 10% to 30% of ATC cases [93,94]. The SWI/SNF complex helps regulate chromatin accessibility and gene transcription by repositioning nucleosomes, thereby controlling the expression of genes tied to cell differentiation, growth, and DNA repair [94,95]. Loss-of-function mutations in these genes disrupt normal chromatin structure. This disruption encourages a more open chromatin state, which supports transcriptional programs linked to stemness and tumour progress [96].

In ATC, mutations in chromatin-remodelling genes, particularly *SWI/SNF* complex subunits such as *ARID1A*, *ARID1B*, *ARID2*, *SMARCB1* frequently co-occur with *TP53* and *TERT* promoter mutations [50]. Loss of *SWI/SNF* function results in repressive chromatin states that reduce thyroid differentiation gene expression, impair radioiodine uptake, and confer resistance to MAPK-pathway redifferentiation strategies; combined with the activation of MAPK, PI3K/AKT, and Wnt/β-catenin signalling, this drives tumour progression and therapeutic resistance [50,96].

Targeting epigenetic regulators offers a promising treatment path for ATC. Preclinical studies have shown that small molecule inhibitors of epigenetic enzymes, like histone deacetylases (HDACs) and bromodomain-containing proteins (BETs) can induce effectiveness in reversing dedifferentiation and restoring response to therapies and can suppress ATC growth [97,98]. However, no epigenetic agent is yet approved for ATC and clinical efficacy remains unproven. Emerging strategies focus on exploiting synthetic-lethal vulnerabilities associated with *SWI/SNF* loss rather than restoring the complex outright [99]. Although there is no direct evidence of synthetic lethality in ATC yet, understanding how epigenetic changes interface with cancer signalling in ATC will be critical for designing rational combination treatments to overcome plasticity and resistance.

### 2.3. Dedifferentiation Trajectory: From Differentiated to Anaplastic

#### 2.3.1. Histological and Molecular Evidence of Progression

ATC represents the most aggressive thyroid malignancy and may arise either de novo or through dedifferentiation from pre-existing DTCs, PTC and FTC. This transformation typically occurs in older individuals, often in the sixth or seventh decade of life, and is marked by the stepwise accumulation of oncogenic alterations across key molecular pathways [3,100].

**Figure 1:**
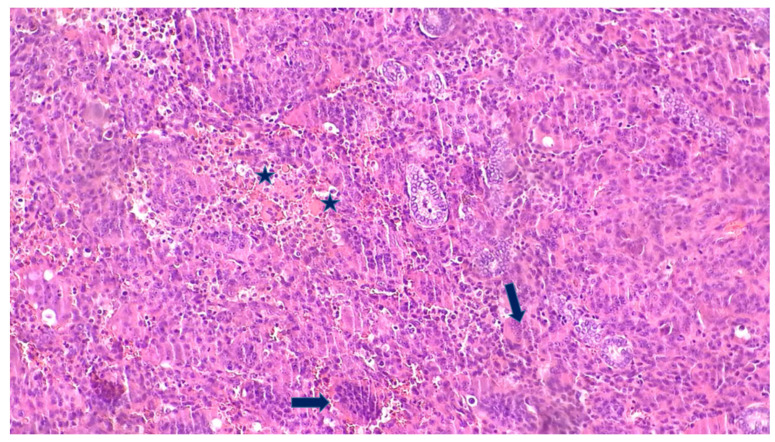
Histological features of anaplastic thyroid carcinoma (ATC) arising in the background of differentiated thyroid carcinoma (DTC), specifically the follicular subtype of papillary thyroid carcinoma (PTC). The residual PTC displays follicular structures lined by tumour cells with optically clear “orphan Annie eye” nuclei and finely dispersed (powdery) chromatin. In the predominant ATC component, arrows indicate multinucleated tumour giant cells, while the stars denote areas of tumour necrosis. Haematoxylin and eosin staining, 10× magnification, captured using an Olympus BX41 microscope with a 10×/22 objective at the Department of Anatomical Pathology, University of Pretoria. The image is derived from anonymised archival diagnostic material and is included for illustrative purposes only.

Histologically, ATCs are frequently associated with residual components of well-differentiated thyroid cancer, suggesting a lineage continuum between DTC and ATC, as shown in Figure 1 [101,102]. Molecular analyses have confirmed that ATCs retain early genetic alterations characteristic of DTCs but acquire additional hits in tumour suppressor pathways such as PTEN-AKT, retinoblastoma (Rb), and TP53, which drive dedifferentiation and tumour progression [25]. During this transformation, tumours lose thyroid-specific functions such as thyroglobulin production and iodine avidity, leading to radioiodine-refractory disease [103,104].

Clinically, dedifferentiation is often recognised during iodine-131 therapy when metastatic lesions fail to concentrate radioactive iodine, despite their presence being confirmed through imaging or histopathological examination [105,106]. Feng et al. (2011) suggested that iodine-131 exposure may further promote dedifferentiation, as demonstrated by the progressive loss of differentiation in FTC-133 cells under long-term in vitro culture [107].

#### 2.3.2. Transitional Role of PDTC

**Figure 2:**
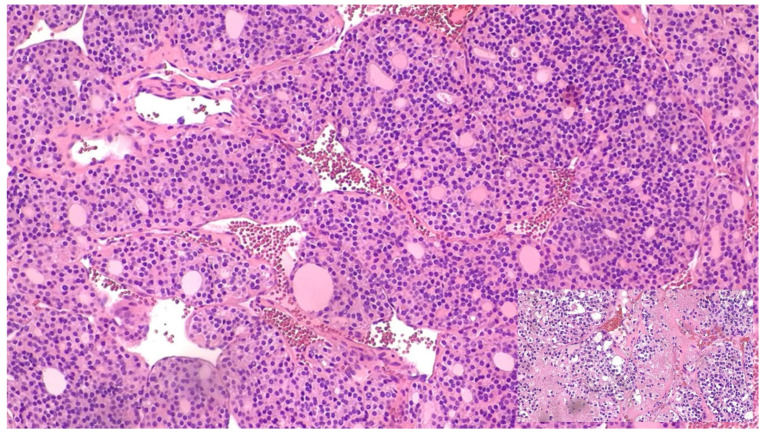
This histological image shows a poorly differentiated thyroid carcinoma (PDTC). The main image shows a characteristic insular (nested) growth pattern (10× magnification). Inset (bottom right) displays a separate tumour field with extensive necrosis (20× magnification). Haematoxylin and eosin staining, images captured at the Department of Anatomical Pathology, University of Pretoria, using an Olympus BX41 microscope with a 10×/22 objective. The image is derived from anonymised archival diagnostic material and is included for illustrative purposes only.

The transition from DTC to ATC may proceed through an intermediate PDTC stage. Histologically, PDTC is characterised by solid, trabecular, or insular growth patterns, increased mitotic activity, and tumour necrosis, but retains some features of follicular cell differentiation—making it morphologically and biologically distinct from both DTC and ATC, as shown in Figure 2. Wen et al. (2019) described a patient in whom a stepwise progression from DTC to PDTC and ultimately ATC was observed. In this case, a rare *TP53* mutation (p.N107_S108delinsX), combined with several low-frequency mutations, appeared to act as a molecular switch for dedifferentiation [108]. These findings suggest PDTC may function as a transitional state, with both genetic and epigenetic events contributing to the dedifferentiation trajectory.

Loberg et al. hypothesised that while second-hit mutations are often implicated in dedifferentiation, the transition from PDTC to ATC may be driven more by changes in the tumour microenvironment than by the acquisition of new oncogenic events [109]. In contrast, large-scale genomic profiling by Landa et al. demonstrated that ATCs harbour significantly higher mutation frequencies in genes such as TP53, TERT promoter, PIK3CA, and components of the SWI/SNF chromatin-remodelling complex compared to PDTCs [50]. Notably, many of these alterations, while present in non-ATC tumours, are often subclonal (low variant allele frequency) in PDTC, but become clonal in ATC, suggesting selective expansion during progression [50]. Loberg et al. used RNA sequencing of 262 thyroid specimens and revealed enrichment of extracellular matrix (ECM)-related and immune activation gene sets in poor-outcome samples. Genes such as *FAP*, *TGFB1*, *COL11A1*, and *WNT2* were highly expressed in ATCs, along with CXCL5, a neutrophil-activating chemokine showing a 43-fold increase. These stromal and immune-related changes were absent in PDTCs, suggesting that a heterogenous fibroblast population and granulocyte infiltration may characterise the ATC microenvironment and work in concert with clonal genomic alterations to drive dedifferentiation [109].

#### 2.3.3. Insights from Mouse Models and Transcriptomics

Robust evidence supporting the multistep dedifferentiation process in thyroid cancer has emerged from genetically engineered mouse models combining oncogenic BRAF V600E expression with TP53 loss. These models faithfully recapitulate the progressive transformation from well-differentiated PTC to PDTC and ultimately ATC [110,111]. The sequential genetic insults in these models, particularly BRAF activation alongside loss of tumour suppressors such as TP53, cooperate to drive tumour dedifferentiation and aggressive phenotypes characteristic of human ATC [50]. Aberrant Wnt/β-catenin signalling has also been implicated in promoting tumour progression within this genetic context.

Functional studies in BRAF V600E/TP53-deficient mice demonstrate that dedifferentiation involves EMT, enhanced proliferation, and acquisition of invasive features, highlighting that tumour plasticity arises from both genetic insults and downstream signalling alterations [110,111].

**Figure 3:**
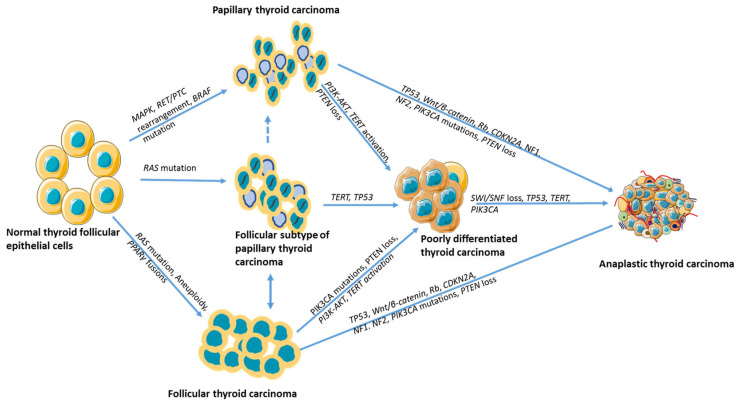
This image illustrates the progression of thyroid follicular epithelial cells to anaplastic thyroid carcinoma (ATC). The transformation is driven by the stepwise accumulation of mutagenic events. Initial mutations such as *RAS*, *BRAF*, *RET/PTC* rearrangements, *PPARγ* fusions, and chromosomal aneuploidy commonly give rise to differentiated thyroid carcinomas (DTCs), including papillary and follicular subtypes. With further genetic alterations, including *TP53* and *RB1* inactivation or *Wnt/β*-catenin pathway activation, DTCs may dedifferentiate into ATC. In some cases, for well-differentiated carcinomas, progression may occur via an intermediate poorly differentiated thyroid carcinoma (PDTC) stage before culminating in ATC through *SWI/SNF*, *TERT* and *TP53* mutations. Transformation of PDTC to ATC is postulated to mutations such as *TP53*, *Wnt/β*-catenin, *Rb*, *FAP*, *TGFB1*, and *COL11A1*.

Collectively, current evidence supports a multistep model of thyroid dedifferentiation in which DTCs undergo progressive transformation to ATC through the accumulation of genetic insults, including TP53, RB1, and Wnt/β-catenin pathway alterations [79]. This process may include a PDTC intermediate. Increasingly, transcriptomic studies and mouse models have revealed that dedifferentiation is not solely governed by mutational burden, but also by transcriptional reprogramming and microenvironmental cues. Notably, factors such as *E2F7*, *p53*, and *Rb* loss have emerged as central regulators of this trajectory, and genes like *FAP*, *TGFB1*, and *CXCL5* underscore the potential role of stromal and immune modulation in supporting tumour plasticity and aggressiveness [109,112]. These findings are summarised in Table 1 and illustrated schematically in Figure 3, which highlights the sequential genetic and microenvironmental changes implicated in the progression from well-differentiated to ATC.

## 3. Cancer Stem Cells in Thyroid Carcinoma

### 3.1. Conceptual Framework and Experimental Evidence

Within the malignant tumour substance, there exists a specific and under-represented subpopulation of tumour cells that exhibit biological and molecular features similar to those of normal stem cells, known as CSCs [114,115]. CSCs were first recognised in acute myeloid leukaemia in 1997 as a subpopulation of tumour-initiating cells originating from primitive haematopoietic cells [116]. Interestingly, it has been suggested that CSCs may explain the extensive intratumoural heterogeneity of diverse cancers [117,118]. CSCs exhibit diverse properties that contribute to the progenitor (or stemness) phenotype, tumour progression, and recurrence, including the ability to self-renew, pluripotency to differentiate into diverse lineages, tumour initiation, clonal long-term repopulation potential, and uncontrolled tumour growth that promotes metastasis and recurrence [119,120]. Moreover, CSCs are more resistant to chemotherapeutic and radiation therapies, making them attractive targets for the development of novel pharmacological agents [118,121,122]. During tumourigenesis, it has been postulated that epigenetic and genetic alterations may deregulate the expression of multiple protein-encoding genes and non-coding RNAs, thereby impacting cancer signalling pathways and resulting in a cell phenotype similar to that of stem cells [123].

CSCs are increasingly recognised as key players in the development, progression, and treatment resistance of thyroid cancer, particularly in ATC. Studies indicate that ATC harbours a higher proportion of CSCs compared to well-differentiated thyroid cancers, contributing to tumour recurrence and metastasis [16,124,125]. Li et al. (2013) identified and enriched CSCs using a spheroid-forming assay, reporting that approximately 3–9% of cells from four ATC cell lines formed thyrospheres. These thyrospheres expressed the stem cell markers NANOG and OCT4 and demonstrated self-renewal capacity. Injection of these cells into the thyroid glands of NOD/SCID Il2rg^−/−^ mice resulted in the formation of metastatic tumours that recapitulated the clinical features of human ATC [126].

Although multipotent stem cells from the adult thyroid gland have not yet been isolated, cells with stem-like properties have been reported in normal thyroid tissue, multinodular goitres, and thyroid cancers. These cells represent a very small fraction of the thyroid follicular cell population (typically <5%) and can be isolated via non-adherent sphere culture in specialised serum-free media [127]. This method, adapted from protocols used to culture progenitor cells in other organs [128], exploits the ability of stem and progenitor cells to survive and form spheres capable of self-renewal while expressing embryonic stem cell biomarkers [129]. Human thyroid stem and progenitor cells cultured in suspension form thyrospheres composed of both stem and progenitor cells. Thyrospheres derived from normal tissue can be induced to differentiate using insulin and insulin-like growth factor [130]. These thyrosphere cells express NANOG and OCT4 and possess self-renewal capacity [131].

### 3.2. CD44 and Other Key Cancer Stem Cell Markers in Thyroid Cancer

**Figure 4:**
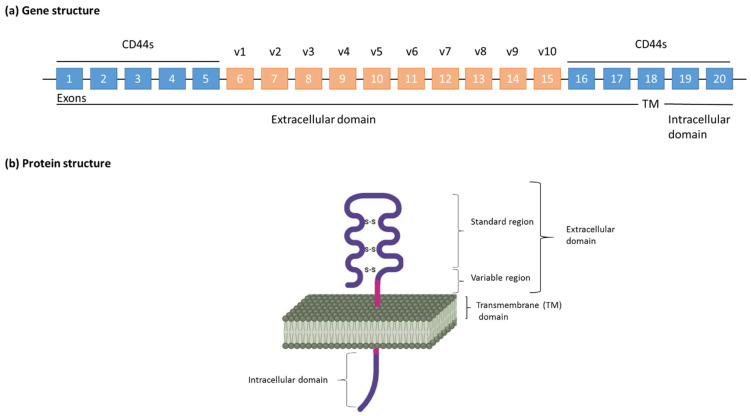
Schematic representation of the gene and protein structure (across the cell membrane) of CD44.

CD44 is a transmembrane glycoprotein encoded by the CD44 gene located on chromosome 11p13 in humans [132]. It plays a multifaceted role in cell adhesion, migration, and intercellular signalling. The gene structure includes 19 exons, with exons 1–5 and 16–20 forming the standard isoform (CD44s), while the central exons (6–15), through alternative splicing, generate multiple variant isoforms (CD44v1-v10), as shown in Figure 4 [133,134,135]. Although numerous splicing combinations are theoretically possible, only a subset has been experimentally validated. These isoforms differ in their extracellular domain composition, which can modulate ligand binding and downstream signalling, contributing to phenotypic diversity within tumour cells [23,136]. In the context of thyroid cancer, particularly ATC, these structural variants may differentially influence stemness and tumour aggressiveness [137,138,139].

CD44 has been widely recognised as a marker of CSCs in several solid tumours, including thyroid malignancies [133,137,140]. In ATC, CD44 expression is frequently accompanied by other stemness-associated proteins such as ALDH1, OCT4, SOX2, and NANOG [141]. CD44^+^ subpopulations isolated from ATC have demonstrated enhanced abilities for self-renewal, proliferation, thyrosphere formation, and tumour initiation in preclinical models [126]. These findings underscore its functional role in sustaining the CSC pool and support its use as a marker to identify and isolate TCSC populations with high tumourigenic potential.

Recent advances in single-cell RNA sequencing (scRNA-seq) have provided high-resolution insights into cellular heterogeneity and stemness-associated programs in aggressive thyroid cancers [142]. Although scRNA-seq studies specifically focused on ATC remain limited, transcriptomic profiling of PDTC has identified distinct tumour subpopulations enriched for stemness, EMT, therapy-resistance signatures, and high expression of CSC-associated markers such as CD44 and ALDH-related genes [50,143]. These findings support the existence of a hierarchically organised tumour cell population and provide molecular validation of CSC-associated biomarkers previously identified using functional and immunophenotypic approaches. As scRNA-seq datasets continue to expand, further refinement of CSC marker specificity and clarification of lineage relationships during dedifferentiation in ATC are anticipated.

One of the key functional aspects of CD44 lies in its interaction with components of the tumour microenvironment, particularly hyaluronic acid (HA) [144]. This interaction facilitates the activation of several intracellular signalling pathways critical for CSC maintenance and survival, notably the PI3K/AKT and MAPK cascades. These pathways are known to regulate processes such as proliferation, resistance to apoptosis, and adaptation to hypoxic and inflammatory niches, which are factors that contribute to the persistence and resilience of CSCs in the harsh tumour microenvironment of ATC [138].

**Figure 5:**
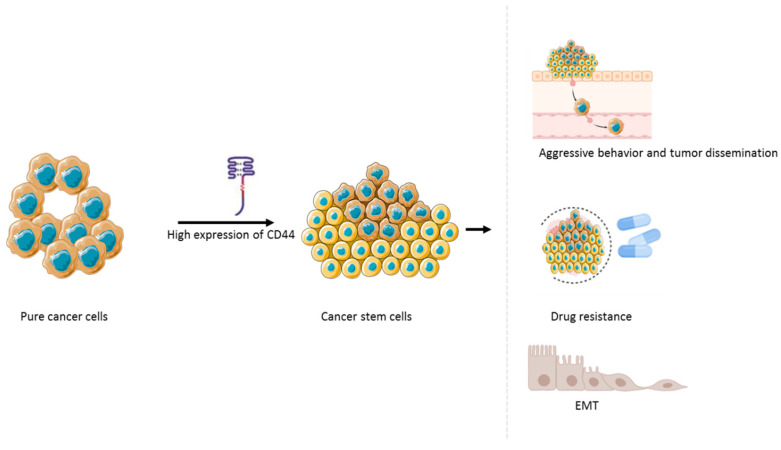
CD44-mediated regulation of cancer cell plasticity. Subpopulations of cancer cells upregulate the iron transporter CD44, which facilitates epigenetic reprogramming and activation of gene expression programs associated with epithelial-to-mesenchymal transition (EMT), stemness, proliferation, and resistance to therapy, which serve as hallmarks of aggressive tumour behaviour.

Beyond its association with stemness, CD44 has been implicated in the promotion of EMT, a process closely linked to dedifferentiation, metastatic progression, and therapeutic resistance, as shown in Figure 5 [145,146,147]. It also supports ferroptosis resistance and EMT-linked iron uptake, facilitating epigenetic reprogramming that reinforces CSC plasticity [148,149]. CD44^+^ cells in ATC often exhibit mesenchymal traits and reduced sensitivity to conventional therapies, including chemotherapy and radiotherapy [138]. Elevated CD44 expression in PDTC and ATC, as shown by immunohistochemistry and flow cytometry, correlates with more aggressive tumour behaviour, increased metastatic potential, and poorer clinical outcomes [137]. These associations support the emerging view of CD44 not merely as a phenotypic marker, but as an active contributor to ATC pathobiology, making it a candidate target for CSC-directed therapeutic strategies.

ATC is associated with a high proportion of CSCs [124]. Another frequently studied CSC marker is CD133, a pentaspan glycoprotein also known as Prominin 1 (PROM1), which localises to cellular protrusions and is expressed in various stem cell populations, including haematopoietic stem cells and endothelial progenitor cells [150,151,152]. Haghpanah et al. (2016) investigated CSC characteristics in ATC cell lines SW1736 and C643 and identified CD133+ subpopulations with elevated expression of ABCG2, CD133, and SOX2, and reduced expression of Nestin, challenging previous assumptions about Nestin as a CSC marker [153].

The CD133+ ATC cell population undergoes self-renewal and can propagate tumours in immunocompromised mice [131]. These cells demonstrate greater radioresistance and higher expression of OCT4 (POU5F1), NANOG, SOX2, LIN28 (LIN28A), and GLUT1 (SLC2A1), along with lower expression of various thyroid-specific genes [154]. In medullary thyroid carcinoma, CD133+ tumour-initiating subpopulations derived from drug-exposed TT cells displayed resistance to 5-FU, maintaining chemoresistant properties during further propagation [155]. Nonetheless, other investigators have not detected CD133 expression in papillary, follicular, or ATC specimens [17]. Alternative CSC markers such as NANOG, OCT4, SOX2, SSEA4, and CD144 have been proposed for characterising CSCs in thyroid cancer tissues [16,131,141].

## 4. The Tumour Microenvironment and Cancer Stemness

**Figure 6:**
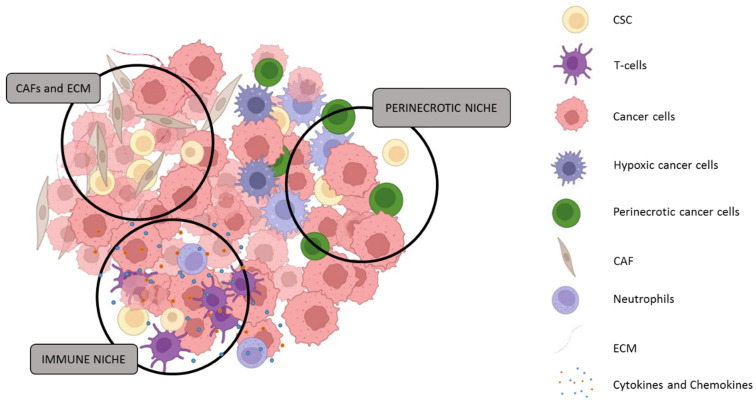
Diagrammatic depiction of the localisation of cancer stem cells (CSCs) within tumour microenvironmental niches. CSCs reside within the extracellular matrix (ECM), which provides structural protection and promotes self-renewal through matrix remodelling. Their localisation within immune niches facilitates immune evasion via immunosuppressive signalling. In perinecrotic regions, CSCs are supported by hypoxia-induced paraphysiological adaptations that enhance survival.

Emerging evidence supports a hierarchical model in ATC, wherein CSCs generate differentiated progeny while maintaining a self-renewing pool of tumour-initiating cells [21,24,124]. The maintenance and plasticity of CSCs are governed not only by intrinsic signalling pathways but also by extrinsic cues from the tumour microenvironment (TME), which plays a central role in sustaining stemness and mediating resistance to therapy [156,157]. Figure 6 summarises the potential localisation of CSCs within specialised niches, namely stromal, immune, and hypoxic microenvironments that promote stem-like features through extracellular matrix remodelling, cytokine signalling, and hypoxia-inducible factor (HIF) activation, thereby supporting CSC survival, adaptation, and therapeutic evasion [16,21,158,159].

### 4.1. CAFs, ECM Remodelling, CD44 Signalling, and Stromal Interactions

Cancer-associated fibroblasts (CAFs) are pivotal components of the tumour microenvironment in ATC, orchestrating extracellular matrix (ECM) remodelling and facilitating CSC maintenance [160]. In thyroid cancer, CAFs secrete key ECM proteins such as collagen I, fibronectin, and osteopontin, which contribute to tumour progression, invasiveness, and resistance to therapy [161,162]. These ECM components not only provide structural support but also serve as bioactive scaffolds that signal through surface receptors on cancer cells to maintain stemness [21].

CD44, a principal cell surface receptor for HA and various ECM proteins, is heavily implicated in these interactions. In thyroid CSCs, CD44 engages with ECM elements such as fibronectin and osteopontin, reinforcing cell adhesion, migration, and survival. Its expression correlates with tumour aggressiveness and therapy resistance [161]. Notably, a subset of CAFs with a pro-stemness phenotype can further amplify CD44-mediated signalling, sustaining CSC populations and even inducing stem-like traits in non-stem cancer cells under stress conditions [118].

These bidirectional interactions, wherein tumour cells activate CAFs and are, in turn, supported by them, create a self-reinforcing loop that promotes tumour progression and stemness [163]. *BRAFV600E*-driven ECM remodelling has also been shown to upregulate CD44, fibronectin, and collagen, linking genetic alterations with stromal dynamics [161]. Collectively, the CAF-ECM-CD44 axis represents a critical niche component in ATC and a potential target for disrupting the CSC-supportive microenvironment.

### 4.2. Immune Microenvironment and Inflammatory Drivers

The immune TME in ATC is characterised by abundant tumour-associated macrophages (TAMs), which may constitute up to 50% of immune infiltrates in advanced tumours [164,165]. TAMs, along with neutrophils and tolerogenic dendritic cells, support CSC survival and invasion. CSCs themselves exhibit low immunogenicity and actively remodel the immune niche to suppress anti-tumour responses [165].

CSC-derived exosomes facilitate immune evasion by promoting TAM polarisation towards an M2 phenotype and suppressing the natural killer (NK) cell function [166]. In turn, TAMs and CAFs release exosomes that enhance EMT, angiogenesis, and stem-like traits in cancer cells. These reciprocal interactions contribute to an immunosuppressive feedback loop that maintains CSC populations and promotes resistance to therapy [167].

### 4.3. Hypoxia and Metabolic Adaptation

Hypoxia is a defining feature of ATC and a crucial regulator of CSC biology. Regions of necrosis and hypoxia are common in ATC and are associated with elevated levels of hypoxia-inducible factors (HIF-1α and HIF-2α), which promote CSC maintenance and therapy resistance [168]. HIFs also upregulate GLUT1 and VEGF, contributing to tumour dedifferentiation and angiogenesis [169]. Notably, HIF-1α may also be activated under normoxic conditions through oncogenic signalling via the PI3K/AKT/mTOR and MAPK (RAS/BRAF) pathways, both commonly dysregulated in ATC [170,171]. This normoxic stabilisation of HIFs amplifies CSC traits and adds a layer of complexity to therapeutic targeting.

In summary, the tumour microenvironment plays a central role in sustaining cancer stemness in ATC. CAF-driven ECM remodelling, immune cell-mediated feedback loops, and hypoxia-driven HIF signalling all converge to support CSC survival, plasticity, and resistance. Molecules such as CD44, which mediate CSC-ECM and stromal interactions, further reinforce these stemness-supportive networks. Targeting these interconnected pathways offers a promising strategy to disrupt the CSC niche and improve treatment outcomes in this aggressive thyroid malignancy.

## 5. Therapeutic Implications and Emerging Strategies

**Figure 7:**
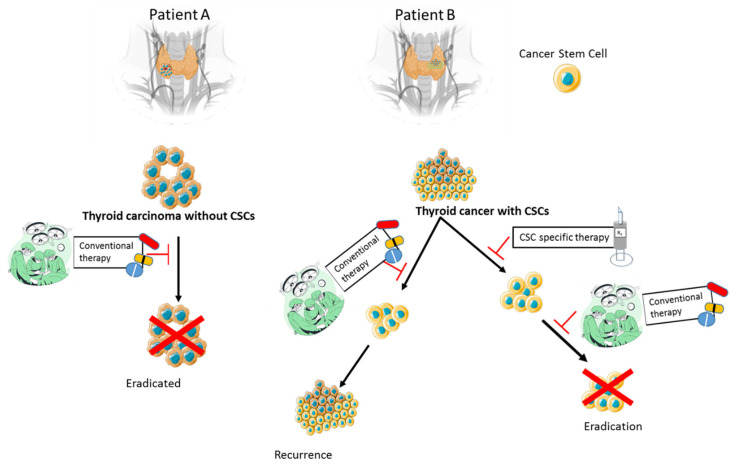
Therapeutic resistance in anaplastic thyroid carcinoma (ATC) illustrating two clinical scenarios. Patient A: In the absence of cancer stem cells (CSCs), conventional therapies such as surgery, chemotherapy, and radioactive iodine effectively eradicate tumour cells. Patient B: In the presence of CSCs (depicted as larger cells with blue nuclei and bright yellow cytoplasm), conventional therapy eliminates bulk tumour cells but spares CSCs, leading to relapse and tumour regrowth. The addition of CSC-targeted therapies in Patient B enables elimination of both CSCs and differentiated tumour cells, reducing the risk of recurrence. Red T-bars denote therapeutic inhibition or blockade, red crosses indicate elimination of tumour cell populations and arrows indicate tumour progression or recurrence.

Therapeutic resistance in ATC remains a critical challenge, despite advances in multimodal treatment. Current guidelines recommend targeted therapy primarily for advanced-stage (IVB-IVC) disease. Traditional trimodal approaches, e.g., surgery, radiotherapy, and chemotherapy, remain foundational, but their efficacy is limited in the context of ATC’s aggressive biology, as shown in Figure 7 [13,172]. As discussed in earlier sections, the convergence of cancer stemness, EMT, and microenvironmental reprogramming contributes significantly to treatment failure and disease progression. These insights have redirected therapeutic development toward strategies that target CSC-associated pathways, reverse EMT, and recondition the tumour microenvironment to enhance responsiveness and clinical outcomes.

### 5.1. Resistance Mechanisms Linked to CSCs and EMT

#### 5.1.1. CD44 and ABC Transporter Expression

TCSCs exhibit high expression of CD44, which contributes to multidrug resistance (MDR) through interactions with ATP-binding cassette (ABC) transporters, notably ABCG2 and ABCB1 (P-glycoprotein) [138,159,173]. CD44-mediated resistance involves the activation of PI3K/AKT and MAPK/ERK pathways, which not only promote survival but upregulate ABC transporter gene expression, enhancing drug efflux capacity. This contributes to low intracellular accumulation of chemotherapeutic agents such as doxorubicin and paclitaxel [174]. Furthermore, CD44 binds HA, triggering downstream Rho GTPase and Ras pathway signalling, enhancing motility, stemness, and therapy evasion [175].

#### 5.1.2. Resistance to Conventional Chemoradiotherapy

The quiescent state of CSCs enables escape from DNA-damaging agents that primarily target proliferating cells [176]. High expression of DNA damage response (DDR) proteins such as RAD51 and CHK1 further supports repair and survival after radiotherapy [177]. Additionally, EMT, often driven by TGF-β, IL-6, or hypoxia-inducible factor-1α (HIF-1α), promotes resistance via activation of transcription factors such as Snail, ZEB1, and TWIST1. These repress E-cadherin and induce mesenchymal markers like vimentin, which facilitate invasion and chemoresistance [177]. EMT also enhances expression of stemness regulators including SOX2, OCT4, and NANOG, creating a feedback loop between EMT and CSC maintenance [118,178].

### 5.2. Targeting Stemness-Associated Pathways

#### 5.2.1. Wnt/β-catenin, Notch, Hedgehog, PI3K/AKT/mTOR

The Wnt/β-catenin pathway plays a pivotal role in maintaining the self-renewal of TCSCs. Aberrant activation, often via downregulation of APC or overexpression of Wnt ligands (e.g., WNT1, WNT10B), leads to nuclear translocation of β-catenin, which partners with TCF/LEF to upregulate genes such as *MYC*, *CCND1*, and *AXIN2*. Inhibitors like ICG-001, which disrupt β-catenin/CBP interaction, have shown efficacy in preclinical thyroid cancer models [179].

Notch signalling, mediated through ligand-receptor interactions (e.g., DLL1/4 and NOTCH1–4), regulates TCSC fate decisions [180]. In ATC, increased NOTCH1 expression correlates with upregulated HES1 and HEY1 expression, contributing to survival and radioresistance [131]. Gamma-secretase inhibitors (GSIs), such as RO4929097, block NICD release and have demonstrated synergistic effects with kinase inhibitors [181].

The Hedgehog pathway, involving SHH-PTCH1-SMO-GLI axis activation, maintains stemness and EMT [182]. Overexpression of GLI1/2 in ATC has been associated with enhanced tumourigenicity [183]. SMO antagonists like vismodegib and GLI inhibitors such as GANT61 suppress sphere formation and CSC marker expression [184,185].

The PI3K/AKT/mTOR pathway is frequently activated in thyroid cancer via PIK3CA mutations or PTEN loss [186]. mTORC1/2 activation promotes metabolic reprogramming and resistance [187]. Dual PI3K/mTOR inhibitors (e.g., BEZ235) or AKT inhibitors (e.g., MK-2206) impair thyroid CSC proliferation and sensitize them to chemotherapy [188,189].

#### 5.2.2. EMT Reversal Agents and Pro-Differentiation Strategies

Targeting EMT involves inhibition of TGF-β signalling (e.g., with SB431542), blockade of IL-6/JAK/STAT3 axis (e.g., with ruxolitinib), and suppression of transcription factors such as TWIST1 and ZEB1 via siRNA or CRISPR-based approaches [190,191,192,193]. Pro-differentiation agents such as histone deacetylase (HDAC) inhibitors (e.g., suberoylanilide hydroxamic acid) and retinoic acid analogues restore thyroid-specific gene expression, including sodium-iodide symporter (NIS), improving radioiodine uptake [194,195]. Further targeted therapeutic strategies against cancer stemness and dedifferentiation in ATC are summarised in Table 2.

### 5.3. Targeting CD44 and CSC-Associated Networks

CD44v6 and CD44v9 isoforms, associated with thyroid tumour aggressiveness, are promising therapeutic targets. Monoclonal antibodies (e.g., BIWA-4 targeting CD44v6) and scFv fragments have been developed, though glycosylation and splicing variability complicate clinical application [139,196]. Antibody-drug conjugates (ADCs) and chimeric antigen receptor (CAR)-T cells engineered against CD44 isoforms are under exploration in preclinical models [197,198].

Interfering with CD44-HA interaction using hyaluronidase or HA oligomers reduces CSC viability and impairs downstream ERK/AKT activation [199]. Silencing CD44 with siRNA or shRNA suppresses expression of EMT markers (e.g., vimentin, N-cadherin), stemness factors, and ABC transporters, thereby restoring drug sensitivity [134,200]. Additionally, blockade of downstream pathways such as PI3K/AKT, RhoA/ROCK, and STAT3 abrogates CD44-driven stemness and invasiveness [136,201].

CD44-targeted imaging is progressing through novel platforms such as [^89^Zr]-labelled immunoliposomes and nanobodies directed against CD44v6 [202,203]. Pretargeted immuno-PET using bispecific antibodies and bioorthogonal tetrazine-ligand chemistry enhances specificity [204,205,206]. CD44 expression profiling through PET/SPECT imaging could guide personalised therapy, particularly in aggressive subtypes like ATC.

### 5.4. Novel Agents and Combination Therapies

#### 5.4.1. Epigenetic Modulators, CDK Inhibitors, siRNA Nanotherapy

HDAC inhibitors (e.g., panobinostat, VPA) induce histone acetylation, re-express silenced thyroid differentiation genes, and downregulate stemness markers [207,208,209]. DNA methyltransferase inhibitors (e.g., decitabine) also reverse silencing of tumour suppressor genes [210]. CDK4/6 inhibitors (e.g., palbociclib) suppress cell cycle progression in TCSCs and show synergy with BRAF or PI3K inhibitors [211].

siRNA-loaded lipid nanoparticles targeting key stemness drivers such as SOX2, OCT4, or CD44 have demonstrated efficacy in vitro and in vivo. These systems improve delivery efficiency, reduce off-target effects, and enable combinatorial approaches with kinase inhibitors or immunotherapy [212,213].

#### 5.4.2. Immunotherapy: Checkpoint Blockade, TIME Modulation

The tumour immune microenvironment (TIME) in ATC is often immunosuppressive, with increased PD-L1, TGF-β, and MDSCs [214,215]. CSCs may contribute to immune evasion by upregulating PD-L1 and reducing MHC-I expression. Immune checkpoint inhibitors (ICIs) targeting PD-1/PD-L1 or CTLA-4 can reverse T cell anergy and promote tumour rejection [216,217]. Combinations with agents targeting CSC pathways (e.g., STAT3 inhibitors or CD44-directed therapy) may improve efficacy [218,219,220].

TIME modulation using CXCR4 antagonists (e.g., plerixafor) enhances T cell infiltration and disrupts the CSC niche, thereby weakening the protective environment that CSCs exploit [221]. Inhibition of IDO, a tryptophan-catabolising enzyme elevated in CSC-rich environments, also holds promise for reshaping the immune contexture [222,223,224,225]. These emerging strategies highlight the crucial intersection between CSC biology and immunotherapy in ATC and justify the focus on immune-targeting agents, as summarised in Table 3.

#### 5.4.3. Multikinase and CSC-Sensitising Agents

Agents targeting multiple tyrosine kinases, including lenvatinib and sorafenib, have demonstrated limited but notable efficacy in ATC [226,227,228]. However, intrinsic resistance driven by CSCs requires combination regimens. CSC-sensitising agents such as metformin, disulfiram (ALDH inhibitor), and salinomycin impair mitochondrial respiration and reduce CSC frequency [229,230,231]. Combining these with multikinase inhibitors may eliminate both bulk tumour cells and resistant CSC populations.

**Table 2:** Targeted therapeutic strategies against cancer stemness and dedifferentiation in anaplastic thyroid carcinoma (ATC).

Therapeutic Focus/Immune Feature	Description/Strategy	References
Cancer Stem Cell (CSC) Targeting	THZ1 (CDK7 inhibitor) suppresses CSC activity, induces apoptosis and reduce self-renewal capacity. THZ531 (CDK12/13 inhibitor) leads to cell cycle arrest and impairs CSC transcriptional programs.	[232,233,234]
EMT and CSC Plasticity Inhibition	Agents reversing EMT include herbimycin A (via PI3K/AKT and TP53) and suberoyl bis-hydroxamic acid (via Notch1/TP53), promoting differentiation and apoptosis of CSC-like cells. Inhibition of TGF-β signalling (e.g., SB431542) and blockade of IL-6/JAK/STAT3 axis (e.g., ruxolitinib) suppress key EMT transcription factors TWIST1 and ZEB1, thereby reducing CSC plasticity and invasiveness.	[68,235,236]
RTKs and Angiogenesis Inhibitors	Multi-kinase inhibitors (sorafenib, lenvatinib, pazopanib) inhibit growth/angiogenesis. Sorafenib combined with metformin or HNHA shows anti-CSC synergy effects in targeting CSC populations. Anlotinib targets CXCL11–EGF–EGFR loop, limiting CSC niche support.	[228,229,237,238]
BRAFV600E Targeting	BRAFV600E seen in ~40% of ATC. BRAF inhibitors (dabrafenib, vemurafenib, PLX4720) reduce proliferation, but monotherapy may enhance CSC traits. Combination strategies are preferred to overcome CSC-mediated resistance and plasticity.	[39,68,239,240]
PI3K/AKT/mTOR Pathway Inhibition	Agents such as 17-AAG, Torin2, and everolimus reduce tumour burden and CSC survival in preclinical models. Combination treatments with metformin and pioglitazone/docetaxel modulates PI3K-AKT-FOXO1 and mTOR pathways crucial for CSC maintenance and resistance to apoptosis.	[241,242,243]
Epigenetic Therapy	HDAC inhibitors (belinostat, panobinostat, VPA) reverse CSC-associated epigenetic modifications, including Panobinostat induces NIS expression and enhancing CSC sensitivity to paclitaxel/doxorubicin activity.	[68,207,244,245]
Additional Molecular Targets	ALK inhibitors (e.g., crizotinib) suppress oncogenic signalling that supports CSCs. TERT-targeting siRNA nanoparticles reduce CSC immortality and self-renewal capacity in preclinical studies.	[246,247]

**Table 3:** Immunological landscape and immunotherapeutic targets in anaplastic thyroid carcinoma (ATC).

Immune Feature	Description/Strategy	References
Tumour Immune Microenvironment	ATC shows macrophage and CD8^+^ T cell infiltration, but cytotoxicity is diminished due to immune exhaustion and M2 polarization. M2-like TAMs accelerate metastasis and increase ATC stemness through secretion of IGF, which activates the IR-A/IGF1R–PI3K/AKT/mTOR signalling pathway.	[248,249]
Checkpoint Molecule Expression	Elevated PD-1, PD-L1, LAG-3, HAVCR-2, TIGIT. Higher PD-L1 in ATC versus PDTC; linked to poorer prognosis. CSCs exploit PD-L1 upregulation to evade immune surveillance, maintaining tumour-initiating potential.	[68,165,250]
Tumour-Associated Macrophages (TAMs)	CD68^+^CD163^+^ TAMs form networks; high density correlates with poor prognosis. Gene markers: FZD6, RBBP8, PREX1, HSD3B7; CXCR4 also implicated. TAM-derived cytokines and IGF signalling reinforce CSC niches and drive ATC progression.	[21,251,252,253]
Immune Genetic Signature	CREB3L1 modulates EMT and immune function; acts as upstream regulator in ATC. CREB3L1 target genes are synchronised with ATC progression and implicated in EMT and mTORC1 pathways. EMT enhances CSC plasticity, while CREB3L1 positivity correlates with relapse, underscoring its role in CSC fate determination.	[254,255]
PD-1/PD-L1 Targeting	Clinical evidence supports use of atezolizumab; dual inhibition of BRAFV600E and PD-L1 enhances TAM recruitment and therapeutic nanoparticle uptake. PD-1/PD-L1 blockade can reduce CSC immune evasion and sensitise CSCs to combined targeted therapy.	[68,242,256,257]
Beyond PD-1: Emerging Targets	Dysfunctional NK cells exhibit reduced cytotoxicity in ATC; radioimmunotherapy and NK reactivation are under exploration. NK cells preferentially eliminate CSCs; restoring NK activity may improve CSC clearance and prevent relapse.	[16,258,259,260,261]

Despite compelling preclinical evidence, the clinical translation of CD44- and CSC-targeted therapies remains challenging. The wide expression of CD44 in normal epithelial and immune cells raises concerns regarding on-target and off-tumour toxicity, limiting therapeutic windows. In addition, extensive isoform heterogeneity and context-dependent signalling complicate patient stratification and therapeutic targeting. CSC plasticity, whereby non-CSC populations can reacquire stem-like properties under therapeutic pressure, further undermines durable responses [22,262]. To date, CSC-directed agents have shown limited success in clinical trials when used as monotherapies, raising the need for combinatorial strategies that integrate CSC targeting with conventional, molecularly targeted, or immunotherapeutic approaches [131]. Addressing these challenges will be critical for translating CD44-focused strategies into clinically meaningful therapies for ATC.

## 6. Future Directions

Dedifferentiated thyroid cancers exhibit unique expression profiles of stromal markers and predicted immune cell infiltrates. Single-cell resolution technologies will be instrumental in clarifying these patterns and elucidating the signalling cues that sustain ATC aggression [55,215]. Importantly, the distinct stromal and immune differences between PDTCs and ATCs in Loberg et al. 2021’s cohort suggests that these tumours may represent pathologically distinct endpoints, rather than existing along a linear dedifferentiation spectrum [109]. This has significant implications for tailored therapeutic strategies.

Advances in molecular profiling and imaging further highlight the growing potential of CSC-targeted diagnostics and therapies in ATC. The development of robust ATC-specific CSC models, together with adaptation of imaging strategies refined in other malignancies, may facilitate earlier detection of treatment-resistant clones and enable real-time monitoring of therapeutic response [200,263,264]. As stemness and EMT remain major barriers to treatment efficacy, multimodal strategies that integrate CSC-targeting agents with immunotherapy and receptor tyrosine kinase inhibitors hold promise for overcoming therapeutic resistance [139,219,245].

Within this context, CD44 emerges as a central mediator of CSC plasticity, EMT, and the tumour–stromal interaction in ATC (Okada et al., 2014; Chen et al., 2018; Mortensen et al., 2023 [134,137,139]). Beyond its role as a diagnostic biomarker, CD44 represents a potential therapeutic target within this highly aggressive subtype. Therapeutic strategies directed at CD44 encompass both direct and indirect approaches. Direct targeting of CD44, particularly variant isoforms such as CD44v6 and CD44v9, has been explored using monoclonal antibodies, antibody–drug conjugates, and nanocarrier-based delivery systems; however, clinical translation has been constrained by isoform heterogeneity and expression in normal tissues [23,138].

Consequently, indirect targeting strategies have gained increasing attention, including disruption of the HA-CD44 axis and inhibition of downstream signalling pathways such as PI3K/AKT, MAPK, STAT3, and xCT/SLC7A11-mediated ferroptosis resistance [147,265]. In addition, CD44-driven interactions with ATP-binding cassette transporters contribute to MDR, providing a strong rationale for combination therapies that integrate CD44 pathway modulation with conventional cytotoxic agents, targeted therapies, or immunotherapy [266].

The above approaches position CD44 as a functional therapeutic node within CSC-associated networks rather than a standalone target, reinforcing the need for combinatorial and context-specific treatment strategies in ATC. Future therapeutic efforts will likely require stratification based on CD44 isoform expression, alongside integration of CD44-targeted strategies with microenvironmental and immune-modulating therapies to effectively disrupt CSC-driven resistance.

Further research is warranted to characterise predictive biomarkers of response, delineate mechanisms of resistance, and translate preclinical insights into clinically actionable interventions. The convergence of CSC biology, immune regulation, and microenvironmental dynamics may pave the way for personalised, more effective treatment approaches for patients with this lethal malignancy.

## 7. Conclusions

ATC remains one of the most rapidly progressive human malignancies that carries a poor prognosis due to its refractoriness to therapy. In the centre of ATC biology, the emergence of CSCs and dedifferentiation processes is a network of genetic alterations, transcriptional reprograming and microenvironmental crosstalk [58,123,160,214,267]. Canonical mutations such as those affecting BRAF, TERT, TP53, and PIK3CA, and dysregulation of signalling axes such as PI3K/AKT/mTOR, MAPK, Wnt/β-catenin, Hedgehog, and Notch reinforce CSC plasticity and ATC progression [25,58,76,268].

CSC-associated markers, particularly CD44 and its variant isoforms, emerge as diagnostic and prognostic tools, and functional mediators of EMT, therapeutic resistance, and tumour–stromal interactions [135,269]. Engagement of CD44 with HA and ECM proteins such as osteopontin and fibronectin activates downstream signalling cascades that support CSC survival, therapy evasion, and niche adaptation [134,270]. These interactions are further sustained by tumour-intrinsic pathways and extrinsic modulators such as CAFs, immune infiltrates (e.g., TAMs), and hypoxia-driven HIF signalling, all of which contribute to a permissive CSC-supportive microenvironment [221,271].

Therapeutically, targeting stemness-related pathways and their downstream effectors holds substantial promise. The development of CSC-targeting strategies ranging from CD44-directed therapeutics, pathway inhibitors (e.g., PI3K, mTOR, GLI), epigenetic modulators, and CSC-specific imaging probes suggests a paradigm shift toward precision and combinatorial approaches [15,55,138,153,272]. Importantly, addressing the plasticity of CSCs and their dynamic interplay with the tumour microenvironment may overcome the limitations of current therapies.

Taken together, integrating molecular, immunologic, and stemness-related insights provides a holistic framework for understanding ATC pathogenesis. As we advance into an era of personalised oncology, therapeutic strategies that disrupt the CSC niche, attenuate dedifferentiation signals, and re-sensitise tumours to conventional and immune-based therapies offer the most promising path forward in altering the natural course of this lethal thyroid malignancy.

## Figures and Tables

**Table 1:** Summary of features and key study findings in the dedifferentiation from differentiated thyroid carcinoma (DTC) to anaplastic thyroid carcinoma (ATC).

Study	Key Molecular Features	Histopathological Observations	Experimental/Functional Findings	Key References
Transition to ATC	Accumulation of secondary mutations: TP53, PTEN, TERT, RB	Loss of follicular architecture; spindle, giant or squamoid cells	Loss of thyroglobulin, TTF-1, and NIS expression; non-iodine avid	[107,108]
Role of E2F7 in ATC	Upregulation of E2F7, a non-canonical master transcription factor	Associated with highly aggressive histology in ATC	Knockdown of E2F7 lead loss of proliferation, migration, and clonogenicity in ATC cell lines	[112]
Stepwise Progression: DTC to PDTC to ATC	TP53 p.N107_S108delinsX mutation as a dedifferentiation ‘switch’	PDTC serves as intermediate state with mixed morphology	Suggests linear progression with accumulation of low-frequency mutations	[108]
Tumour Microenvironment Influence	Upregulation of ECM genes (e.g., FAP, PDPN, POSTN); increase in CXCL5	Increased neutrophil and macrophage infiltration in ATC vs PDTC	Suggests fibroblast heterogeneity and immune infiltration drive ATC aggression	[109]
Radioiodine-induced Dedifferentiation	Long-term exposure to ^131^I may enhance dedifferentiation	Functional loss of iodine uptake	FTC-133 cells showed dedifferentiation in long-term culture with ^131^I	[107]
STRN-ALK Fusion Model	STRN-ALK fusion + p53 loss	Mouse models developed PDTC and progressed to ATC	Demonstrates multistep dedifferentiation and coexistence of differentiated and undifferentiated components	[113]

## Data Availability

No new data were created or analyzed in this study.

## Abbreviations

The following abbreviations are used in this manuscript:

ABC	ATP-Binding Cassette
ADCs	Antibody drug conjugates
AJCC	American Joint Committee on Cancer
ALDH	Aldehyde Dehydrogenase
APC	Adenomatous Polyposis Coli
ASPM	Abnormal Spindle-like Microcephaly-Associated Protein
ATC	Anaplastic Thyroid Carcinoma
AXIN	Axis Inhibition Protein
CAFs	Cancer-Associated Fibroblasts
CAR-T	Chimeric Antigen Receptor T-cell
CDK	Cyclin-Dependent Kinase
CSC	Cancer Stem Cell
CTLA-4	Cytotoxic T-Lymphocyte Antigen 4
CXCR4	C-X-C Motif Chemokine Receptor 4
DTC	Differentiated Thyroid Carcinoma
ECM	Extracellular Matrix
EMT	Epithelial-to-Mesenchymal Transition
ETS	E26 Transformation-Specific
FAP	Fibroblast Activation Protein
FZD	Frizzled
FTC	Follicular Thyroid Carcinoma
GLI	Glioma-Associated Oncogene
GLUT1	Glucose Transporter Type 1
GSK3β	Glycogen Synthase Kinase 3β
HA	hyaluronic acid
HDAC	Histone Deacetylase
HES/HEY	Hairy/Enhancer of Split / HES-Related with YRPW Motif
HIF	Hypoxia-Inducible Factor
ICIs	Immune Checkpoint Inhibitors
IDO	Indoleamine 2,3-Dioxygenase
IL-6	Interleukin-6
JAK/STAT	Janus Kinase / Signal Transducer and Activator of Transcription
LMICs	Low- and Middle-Income Countries
MAPK	Mitogen-Activated Protein Kinase
MDR	Multidrug Resistance
MDM2	Mouse Double Minute 2 Homolog
mTOR	Mammalian Target of Rapamycin
NIS	Sodium-Iodide Symporter
NK	Natural Killer Cells
NOD/SCID	Non-Obese Diabetic/Severe Combined Immunodeficient
PD-1	Programmed Cell Death Protein 1
PD-L1	Programmed Death Ligand 1
PDTC	Poorly Differentiated Thyroid Carcinoma
PET/SPE	Positron Emission Tomography/Single-Photon Emission Computed Tomography
PI3K	Phosphoinositide 3-Kinase
PTC	Papillary Thyroid Carcinoma
RNA-seq	RNA Sequencing
RTK	Receptor Tyrosine Kinase
SCID	Severe Combined Immunodeficient
shRNA	Short Hairpin RNA
siRNA	Small Interfering RNA
SMO	Smoothened
SNAIL, ZEB1, TWIST1	EMT-Associated Transcription Factors
SWI/SNF	SWitch/Sucrose Nonfermenting
TAMs	Tumour-Associated Macrophages
TCSC	Thyroid Cancer Stem Cell
TGF-β	Transforming Growth Factor Beta
TIME	Tumour Immune Microenvironment
TME	Tumour Microenvironment
TTF-1	Thyroid Transcription Factor-1
VEGF	Vascular Endothelial Growth Factor
Wnt	Wingless-Related Integration Site
